# Association between parental factors and child's behaviors during moderate sedation in pediatric dental care

**DOI:** 10.3389/fped.2024.1456395

**Published:** 2024-09-03

**Authors:** Mohamad A. Alanbari, Hebah M. Hamdan, Omar A. Bawazir, Ayman M. Sulimany

**Affiliations:** ^1^ Department of Pediatric Dentistry and Orthodontics, College of Dentistry, King Saud University, Riyadh, Saudi Arabia; ^2^ Pediatric Dentistry Department, Prince Sultan Military Medical City, Riyadh, Saudi Arabia; ^3^ Department of Periodontics and Community Dentistry, College of Dentistry, King Saud University, Riyadh, Saudi Arabia

**Keywords:** child behavior, conscious sedation, coping skills, catastrophization, dental anxiety

## Abstract

**Background/Aim:**

Managing young children with negative behaviors can be challenging in dental settings. Moderate sedation (MS) is often used as a treatment option for such children. However, children's behavior during MS may vary depending on several variables. These variables include parental factors, such as parental anxiety, coping strategies, and pain catastrophizing. However, this area, particularly in Saudi Arabia, remains underexplored. Therefore, this study aimed to assess the association among parental anxiety, coping style, pain catastrophizing, and children's behavior during MS among Saudi children.

**Methods:**

Based on sample size calculation, this cross-sectional observational study included 85 children aged 3–5 years undergoing dental treatment under MS at King Saud University, Riyadh, Saudi Arabia. Parental anxiety, coping styles, and pain catastrophizing were assessed using the Modified Dental Anxiety Scale, Brief Coping Orientation to Problems Experienced Scale, and Pain Catastrophizing Scale. Child behavior was evaluated using the Houpt scale during sedation visits, which was video-recorded and independently analyzed by a single evaluator. Data were analyzed using Pearson's chi-squared test, Mann–Whitney U test, and stepwise multivariate logistic regression analyses.

**Results:**

The results showed no significant association among parental dental anxiety, pain catastrophizing, and child behaviors during MS. Specific parental coping strategies, such as acceptance, were positively associated with positive sedation outcomes (*P* = 0.03), while active coping strategies were linked to less favorable outcomes (*P* = 0.03). Female children had higher sedation failure rates (*P* = 0.02), and the number of dental treatments was positively associated with success rates (*P* = 0.03).

**Conclusion:**

Parental anxiety and pain catastrophizing did not significantly affect the success of sedation. However, acceptance as a coping strategy was significantly associated with sedation success in pediatric dental care under MS, whereas active coping strategies were associated with less favorable outcomes.

## 1 Introduction

The dental care of young children with negative behaviors is challenging, as disruptive behaviors can complicate and prolong routine dental care, thereby requiring extra resources to accomplish effective treatment (1, 2). Children who lack psychological maturity or have unpleasant experiences at dental clinics may be indicated for advanced behavioral guidance techniques (3). In such situations, the use of advanced behavioral guidance techniques, such as moderate sedation (MS), to provide dental rehabilitation can be a valid option (3). The American Academy of Pediatric Dentistry (AAPD) proposes different levels of intended sedation: minimal, moderate, and deep (4). MS is defined as “a drug-induced depression of consciousness during which patients respond purposefully to verbal commands or after light tactile stimulation” (4). MS is an excellent option for reducing fear and anxiety in children, especially when basic behavioral guidance is unsuccessful (3). However, according to Nelson and Xu (5), a number of factors play crucial roles in successful dental rehabilitation under MS (5). During MS, a child's behavior can vary depending on several factors, including medicine and route (6–8), sex (9), age (10, 11), drug regimen (12, 13), and temperament (10, 14). In addition, parental factors, such as anxiety, coping style, and pain catastrophizing, may affect the success of MS (15).

Parental anxiety is positively associated with dental fear and anxiety in children (16, 17). A study conducted in Saudi Arabia on family factors and dental fear found that children of anxious mothers were more fearful than those whose mothers had no dental fear (18). There is a paucity of research on the prevalence of dental anxiety among children in Saudi Arabia (19, 20)**.** Alshuaibi et al. found that 50.4% of boys and 71. 28% of girls in Al Ahsa had high levels of dental anxiety (19). Additionally, another study done in Riyadh revealed that 28.5% of younger individuals and female participants showed increased anxiety levels (20). Dental anxiety and fear are associated with lower oral health-related quality of life in children and oral care neglect, which exacerbates pre-existing dental issues (21, 22). This neglect leads to more complicated and painful treatments and, therefore, worsens the patient's initial anxieties, leading to a cycle of dental care avoidance and worsening of relationships with dental professionals (23–26). Coping strategies are actions that individuals take to deal with the stress, demands, and conflicts in daily life (27). These coping mechanisms are essential for individuals and have a considerable effect on family dynamics, particularly between parents and children. For instance, a study conducted to predict children's responses to invasive medical procedures found an association between parents’ coping and distress behaviors and their own coping and distress responses (28). Given the prevalence of dental anxiety in children, understanding the dynamics of parental coping styles is crucial (29). Another factor that demands attention when examining children's behavior during MS in dental settings is parental pain catastrophizing, which is defined as having an exaggerated negative mental set brought to bear during actual or anticipated painful experience (30). It is a multidimensional construct, with several theoretical frameworks explaining its mechanisms (31)**.** Appraisal theory suggests that pain catastrophizing arises from primary appraisals (initial evaluations of stressors) and secondary appraisals (assessments of coping strategies and their potential success) (31). Attention bias theory posits that pain catastrophizers focus excessively on pain-related stimuli, similar to patterns observed in anxiety and depressive disorders (32)**.** This heightened focus aligns with information processing theory, which posits that pain catastrophizing affects how sensory and emotional pain information is processed (31). The communal coping model views pain catastrophizing as a coping strategy to gain emotional or tangible support from others (31, 33). The neural underpinnings of pain catastrophizing involve increased activity in brain regions responsible for processing pain's emotional aspects, such as the anterior cingulate and prefrontal cortex (31). The central nervous system (CNS) mechanisms behind this include changes in processes like enhanced temporal summation and disruptions in the hypothalamic–pituitary–adrenal axis, which exacerbate pain perception (31)**.** Despite extensive research in medical contexts, there is insufficient examination of pain catastrophizing within the dental field. Understanding these mechanisms is crucial, as they can affect how parents perceive and respond to pain, potentially increasing their children's sensitivity to pain and catastrophizing behaviors (34, 35).

De Castro Morais Machado et al. (15) conducted a study in Brazil that examined the effects of specific parental factors, including anxiety, coping style, and parental pain catastrophizing, on the behavior of children during MS. The authors found that parental adaptive coping strategies, specifically acceptance and planning, had a positive effect on the behavior of children with MS. However, parental dental anxiety and pain catastrophizing were not related to MS (15). However, there is a lack of literature evaluating the association between parental factors and the behavior of children during treatment under the standard MS regimen in other societies. Parental factors and its association with child behavior may vary among different cultures and populations. Therefore, this study aimed to evaluate the association between parental anxiety, coping style, and pain catastrophizing and children's behaviors during MS in the Saudi population.

## 2 Methods

### 2.1 Ethical approval

This study was approved on December 6, 2022, by the King Saud University (KSU) Institutional Review Board (E-22-7352) and registered at the College of Dentistry Research Center (No. PR 0151), KSU, Riyadh, Saudi Arabia. Informed consent was obtained from the participating parents, including approval for video recording during sedation ensuring transparency and privacy.

### 2.2 Sample calculation and study population

The G*Power program (version 3.1.9.4) was used to calculate the sample size. With an effect size of 0.325, power of 0.90, and level of significance of 0.05, the sample size should include at least 83 patients.

The inclusion criteria for this study were Saudi children aged 3–5 years, with American Society of Anesthesiologists (ASA) I physical status as per the ASA classification (36), and displaying negative behavior on the Frankl Rating Scale (37) during screening in the pediatric department at Dental University Hospital (DUH) at KSU, Riyadh, Saudi Arabia. Eligible participants were required to have no more than two dental treatment visits under MS; have parents who could communicate in Arabic and provided consent to their child's participation; meet the MS physical assessment criteria at DUH in KSU in accordance with AAPD (4); possess no prior experience with general anesthesia (GA), deep sedation, or MS related to dental procedures; and not on a GA waiting list. Additionally, children can voluntarily ingest medication without spitting it.

### 2.3 Study design

This was a cross-sectional observational study of Saudi children who underwent dental treatment for MS between January 2023 and March 2024. This study consisted of two visits, a screening visit and a sedation visit. The screening visit was scheduled at least 1 week before the sedation visit. During the screening visit, a physical assessment was conducted to evaluate the overall health of patients undergoing dental treatment under MS. Subsequently, the questionnaire was distributed to the legal guardians of the children, which consisted of four sections:

(1) Demographic information.
(2) Parental anxiety was assessed using the Arabic version of the Modified Dental Anxiety Scale, which has been validated for assessing dental anxiety (38, 39). This scale assesses anxiety experienced by respondents in response to five different situations faced by patients in the dental clinic: (i) having a dental appointment scheduled for the next day, (ii) being in the waiting area of a dental clinic, (iii) undergoing tooth drilling, (iv) having teeth scaled, and (v) receiving a local anesthetic injection. The total score ranges from 5 to 25 with each question scored using a Likert scale with 5 possible responses: Score 1: not anxious, 2: slightly anxious, 3: fairly anxious, 4: very anxious, and 5: extremely anxious (39). The MDAS has demonstrated good reliability and validity (39).
(3) Parental coping style using the Arabic version of the Brief Coping Orientation to Problems Experienced (Brief COPE), which measures coping strategies**,** was validated for use in this demographic by Alghamdi (40, 41). This scale comprises 28 items that evaluate how individuals cope with stress in their lives, with each item representing a specific coping strategy, including strategies for dealing with dental problems and noncooperation with their children during dental treatment. Parents were surveyed on their strategies and the frequency of engaging in these behaviors. The scale yields 14 subscale scores, each consisting of two items, covering domains, such as active coping, planning, positive reframing, acceptance, humor, religion, emotional support, instrumental support, self-distraction, denial, venting, substance use, behavioral disengagement, and self-blame. Responses were recorded on a four-point scale.
(4) Pain catastrophizing was assessed using the Arabic version of the Parental Pain Catastrophizing Scale validated by Terkawi et al. (42) to assess pain-related thoughts and feelings (30, 42). This scale contains 13 items and uses a 5-point Likert-type scale (0 = not at all, 1 = to a slight degree, 2 = to a moderate degree, 3 = to a great degree, 4 = all the time) to rank the extent to which parents describe their thoughts and feelings when they are in pain. Higher scores indicate a greater tendency towards pain catastrophizing.

### 2.4 Sedation visit

Children's health status, vital signs, chest auscultation, and fasting protocols were thoroughly evaluated to ensure safety and suitability for MS prior to the procedure. The patients were orally administered the sedative agent [midazolam 0.7 mg/kg, at a maximum dose of 20 mg (Hikma Midazolam®), Hikma Farmacêutica, Portugal]. The patients were placed in a dental chair after waiting for 10–15 min. A papoose board was used to protect the child, and a nitrous oxide nasal hood was placed over the child's nose at a ratio of 50/50 and lowered downward at the operator's discretion to maintain an optimal level of sedation. The parents were instructed to leave the clinic and wait in the waiting area. The children were monitored according to the AAPD guidelines for monitoring and managing pediatric patients during and after sedation in clinical procedures (4). All dental treatments were provided by pediatric dentistry residents under the supervision of a consultant in pediatric dentistry. After dental treatment, the patients were administered 100% oxygen for a minimum of 5 min and transferred to a recovery room until they were ready for discharge.

Children's behavior was ranked according to the Houpt scale for overall sedation results and intraoperative behavior to evaluate the participants’ behavior throughout the sedation process (43). An overall Houpt score of good, very good, or excellent was considered successful sedation, and aborted, poor, or fair was considered failure.

All sedation sessions were recorded using a video camera (Canon HFR 806; Canon Inc., Tokyo, Japan) from the administration of nitrous oxide until the time of nitrous oxide removal, to assess the behavior of the children during the first MS by a single independent evaluator (MA) who was blinded to the scores of parental anxiety, coping scales, and pain catastrophizing. A training session was conducted to calibrate the evaluator and analyze the behaviors recorded during the sedation session. Children's behaviors were assessed consistently by analyzing the videos according to predefined criteria (Houpt scale). Ten videos were rated twice by the same evaluator for the intra-examiner reliability analysis. The kappa test showed that the intra-examiner reliability for the assessment of sedation (overall behavior rating scale) was excellent, with a score of 0.96.

### 2.5 Statistical analyses

Data were analyzed using SPSS version 20.0 (IBM Corp., Chicago, IL, USA). Descriptive statistical analysis was applied to demographic data. Pearson's chi-squared test was used to examine the association between children's behaviors and categorical variables, specifically age and sex. The Mann–Whitney U test was used to assess the association between children's behaviors and continuous variables, which included the total number of treatments, parental factors, and the duration of sedation. A stepwise multivariate logistic regression model was used to select the best set of variables to assess the effects of parental factors after controlling for possible confounders. Statistical significance was set at *P* ≤ 0.05.

## 3 Results

### 3.1 Demographics

A total of 85 Saudi children aged 3–5 years and their legal guardians participated in this study. The participants’ demographics were closely divided by sex, with 43 (50.59%) girls and 42 (49.41%) boys (Table 1). The age distribution showed a predominance of 5-year-old children, accounting for 44.71% of the participants. Of the 85 participants, 57 (67.06%) were reported to have successful sedation, whereas 28 (32.94%) were considered to have failed sedations. The average number of dental treatments during the sedation visit was 3.64, and the mean duration of sedation was approximately 22.8 min.

**Table 1:** Demographic and treatment characteristics of patients undergoing dental treatment under moderate sedation.

Variables	*N*	%
Sex		
Male	42	49.41
Female	43	50.59
Distribution of children based on age		
3	21	24.70
4	26	30.59
5	38	44.71
Sedation status		
Success	57	67.06
Failure	28	32.94
Variable	Mean	Standard deviation
Total number of treatments	3.64	1.71
Time of the sedation in minutes	22.8	7.5

### 3.2 Primary outcomes

**Figure 1:**
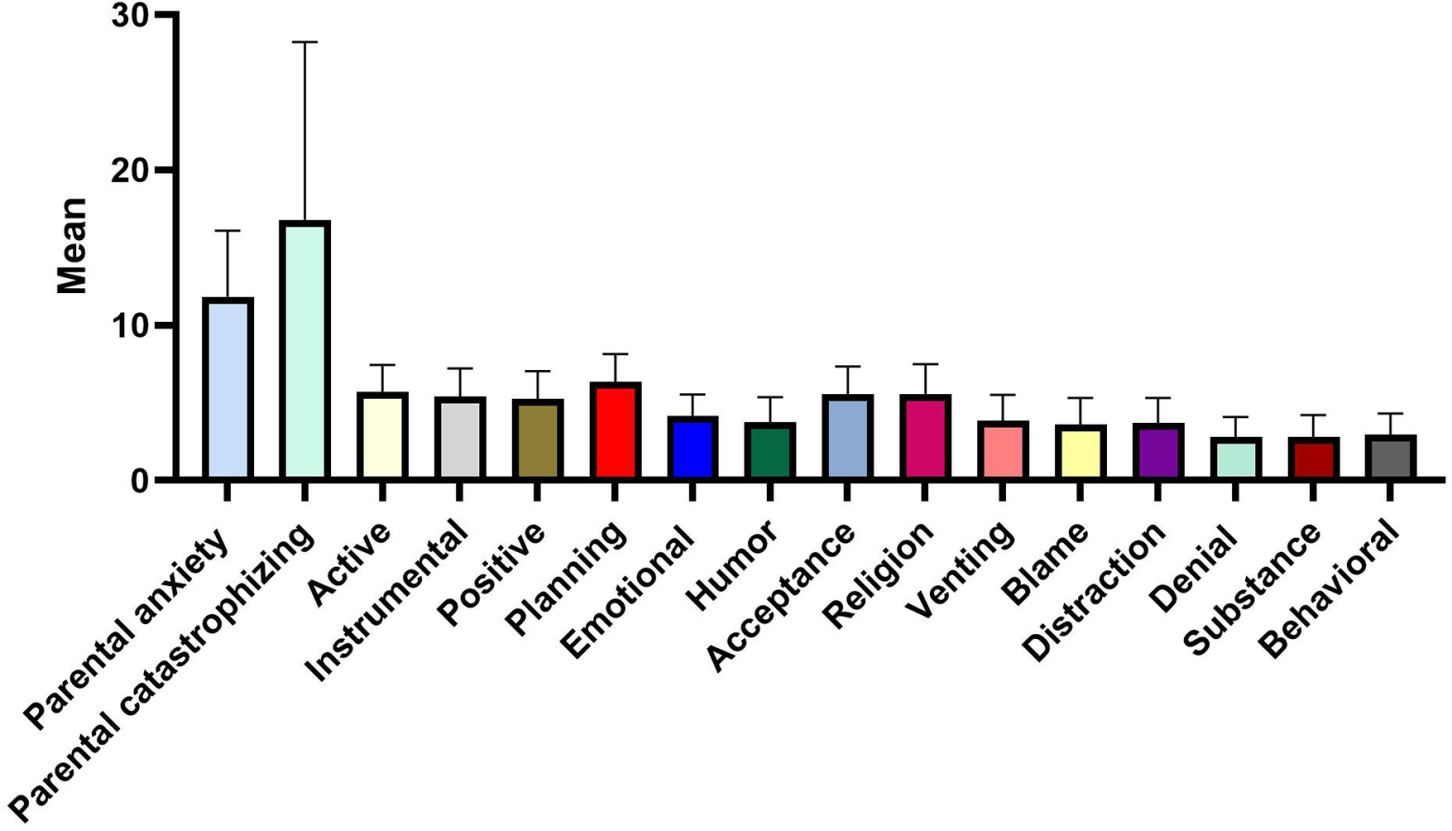
Descriptive (parental factors).

The average scores for parental dental anxiety and pain catastrophizing of the participant group were 11.84 ± 4.24 and 16.78 ± 11.46, respectively (Figure 1). Among the various coping strategies utilized by parents, the strategy of planning and active coping were the most favored approaches, with mean scores of 6.34 ± 1.79 and 5.72 ± 1.73, respectively, whereas the least employed coping strategies were denial and substance use, both with mean scores of 2.81 ± 1.27 and 2.81 ± 1.41, respectively.

The results showed no significant association between parental dental anxiety and MS success (*P* = 0.28) or between parental pain catastrophizing and MS success (*P* = 0.88) (Table 2). Regarding coping style, only acceptance demonstrated a statistically significant association with successful sedation outcomes (*P* = 0.03).

**Table 2:** Parent-related factors and their association with sedation status.

Sedation	Success	Failure	*P* value*
	Median [interquartile range (IQR)]	Median (IQR)	
Parental anxiety	11 (6)	11 (9)	0.28
Parental catastrophizing	14 (14)	16.5 (16)	0.88
Acceptance	6 (3.0)	5 (2.0)	0.03
Instrumental support	5 (3.0)	5 (3.5)	0.50
Active	5 (3.0)	6 (3.0)	0.47
Positive reframing	5 (3.0)	5 (2.5)	0.72
Planning	7 (3.0)	6.5 (3.0)	0.39
Emotional support	4 (2.0)	4 (2.0)	0.74
Humor	4 (3.0)	4 (2.0)	0.99
Religion	6 (3.0)	6 (2.0)	0.39
Venting	4 (2.0)	3 (3.0)	0.22
Blame	3 (3.0)	3 (2.0)	0.33
Self-distraction	3 (2.0)	3 (2.5)	0.54
Denial	2 (1.0)	2 (1.5)	0.64
Substance	2 (2.0)	2 (1.5)	0.84
Behavioral disengagement	2 (2.0)	2 (1.0)	0.22

* Calculated using the Mann–Whitney U test.

### 3.3 Secondary outcomes

Among child-related factors associated with successful sedation, sex was significantly associated with a higher proportion of sedation failures among females (44.19%) than among males (21.43%) (*P* = 0.02). Age-specific patterns did not show significant differences, indicating that behaviors during sedation were consistent across the 3–5 years age range (*P* = 0.50) (Table 3). The association between treatment-related factors (total number of treatments and time) and sedation success was not significant; however, successful patients received more dental treatments over a longer period.

**Table 3:** Child- and treatment-related factors and their association with sedation status.

Variable	Success	Failure	*P* value
	*N* (%)	*N* (%)	
Sex			0.02*
Male	33 (78.57)	9 (21.43)	
Female	24 (55.81)	19 (44.19)	
Patients’ age			0.50*
3	12 (57.14)	9 (42.86)	
4	19 (73.08)	7 (26.92)	
5	26 (68.42)	12 (31.58)	
Number of treatments	Success	Failure	*P* value
	Median [interquartile range (IQR)]	Median (IQR)	
	4 (3.0)	4 (2.0)	0.10**
Time of the sedation	Success	Failure	*P* value
	Median (IQR)	Median (IQR)	
	24 (8.0)	22 (9.0)	0.12**

* Calculated using the Pearson chi-squared test.

** Calculated using the Mann–Whitney U test.

### 3.4 Multivariate analysis

The stepwise multiple logistic regression results showed that parents employing acceptance coping strategies had significantly higher odds of having successful sedation compared with others [odds ratio (OR) = 1.77; 95% confidence interval (CI), 1.19–2.63] (Table 4). Conversely, parents applying active coping was significantly associated with a lower likelihood of having successful sedation (OR = 0.64; 95% CI, 0.44–0.95). Additionally, female patients were significantly associated with a lower likelihood of successful sedation (OR = 0.27; 95% CI, 0.09–0.78), whereas the number of treatments provided was positively associated with higher odds of having successful sedation outcome (OR = 1.47; 95% CI, 1.04–2.08).

**Table 4:** Stepwise multiple logistic regression.

Variable	Odds ratio	95% confidence interval	*P* value
Active coping	0.64	(0.44–0.95)	0.03
Acceptance coping	1.77	(1.19–2.63)	0.005
Sex			
Male	Ref	Ref	0.02
Female	0.27	(0.09–0.78)	
Total number of treatments	1.47	(1.04–2.08)	0.03

## 4 Discussion

Dental treatment of uncooperative children presents considerable challenges for pediatric dentists (44). MS is often employed as a possible option to facilitate dental procedures (3). However, the success of MS is influenced by several factors, including dental (sedation regimen and protocol), childhood (age and sex), and parental factors (15). Given the complexity of parental factors and the limited studies on this topic, this study was conducted to assess the associations among parental dental anxiety, coping styles, and pain catastrophizing in children undergoing dental treatment under MS.

In this study, the results did not show a significant association between parental anxiety and sedation success, which is consistent with the findings of a Brazilian study (15). Previous studies have emphasized the significance of parental anxiety in relation to children's dental fear, pain intensity, and behavior (16–18). This discrepancy may be attributed to the generally low scores of parental dental anxiety reported in this study among the participating parents, indicating that parental anxiety was not a major stressor influencing the child's behavior during MS. Additionally, although parental anxiety may influence the child's initial response to dental settings, its effect on the child's behavior during sedation may be mediated by other factors.

This study highlights the importance of parental coping techniques in pediatric dental sedation, showing a positive association between acceptance and successful outcomes. This aligns with the results of previous studies suggesting that parents using an acceptance-based approach predict improved behavior in children with MS (15, 45). Acceptance is an emotion-focused coping strategy that aims to alleviate emotional conflicts associated with stressful situations, suggesting its effectiveness in helping children navigate the sedation process more effectively (46). Acceptance as a coping strategy might allow parents to better manage their stress, which in turn creates a calmer environment for the child, helping to reduce the child's anxiety and improve sedation outcomes. Conversely, there is no evidence from prior studies that active coping adversely influences sedation outcomes. The unfavorable influence of active coping methods on successful sedation outcomes found in this study requires further investigation. Although active coping is generally considered helpful in stress management, it may increase the awareness of stressors, potentially leading to unintentional disruptive behavior in children. The effectiveness of coping mechanisms is highly context-dependent, supporting the hypothesis of Lazarus and Folkman (27). Lazarus and Folkman proposed that coping strategies are neither universally effective nor ineffective; instead, their effectiveness depends on the situation in which they are deployed and the individual's appraisal of the situation (27).

This study found no significant association between parental pain catastrophizing and sedation success, similar to the findings of a Brazilian study (15). This could be due to the low incidence of high catastrophizing scores in the sample, suggesting that pain catastrophizing is not a significant factor affecting child's behavior during MS. However, other studies have emphasized its importance in children's health outcomes, especially in the context of chronic pain (34, 35).

The notable role of sex in sedation outcomes, with females exhibiting a higher likelihood of sedation failure than males, is consistent with the results of prior studies demonstrating sex differences in pain perception and behavioral responses to dental procedures (9, 10). Furthermore, evidence suggests that sedation is significantly more successful in male than in female children, underscoring the need for sedation approaches that are responsive to sex-specific characteristics (47, 48). Moreover, clinical trials have shown that males may demonstrate greater sensitivity to specific sedatives or analgesic medications than females (49). This highlights the complexity of sex dynamics in pediatric sedation, suggesting the need for further studies to unravel how sedative pharmacodynamics differ between sexes.

The total number of dental treatments completed during sedation sessions was positively associated with the success of sedation. Successful sedation induces a calm state, reduces disruptive behaviors, and allows dental professionals to provide more dental treatment in one sitting while assuring the child's comfort and minimizing psychological suffering.

In this study, all dental factors influencing MS success were controlled by applying standard sedation protocols and similar doses and routes of administration to all children. In addition, the sex distribution among children was nearly equal, minimizing any bias that could arise from sex imbalances. Additionally, all physical assessments and examinations were performed according to the recommendations of the AAPD guidelines to ensure patient safety and provide optimal treatment conditions. Furthermore, trained examiners assessed child behavior during MS using a reliable scale and video camera to enhance the reliability of the results. However, the findings of this study must be interpreted within the context of its limitations. Conducting the research within a single institution limits the generalizability of the findings. The sample is geographically constrained, which may not account for variations in parental behaviors, coping strategies, and children's responses to sedation across different regions and cultural backgrounds. Therefore, future multi-center studies involving different populations are necessary to validate these findings and provide a more comprehensive understanding of the impact of parental factors on children's behavior during sedation. Additionally, the complexity of behaviors during sedation procedures requires further exploration. Children's reactions to sedation are influenced by multiple factors, including their previous medical experiences, temperament, and even the specific interactions with dental staff on the day of the procedure. Further studies are required to explore other parental factors, such as parental presence in the clinic during sedation. Parental presence might provide comfort and reduce anxiety for some children, while for others, it might cause increased stress or interfere with the dental team's ability to manage the procedure effectively. Investigating the impact of parental presence in future research can offer insights into optimizing sedation practices and improving outcomes. Understanding these dynamics may offer valuable insights for optimizing pediatric dental procedures.

Achieving optimal sedation results in pediatric dentistry requires the recognition that the process is multifactorial, including factors such as parental influence, age of children, children's experiences with medical environments, dental teams, and temperament (50). This suggests that a comprehensive approach that incorporates awareness of these dynamics is essential for improving the effectiveness of sedation and patient outcomes. Promoting acceptance-based coping strategies among parents can have significant practical applications in pediatric dental settings. By encouraging parents to adopt these strategies, dental practitioners can help create a more supportive and calmer environment, which may improve sedation outcomes for children.

## 5 Conclusion

Parental anxiety and pain catastrophizing were not significantly associated with sedation success. However, acceptance as a coping strategy was the only significant factor positively associated with successful sedation outcomes. Conversely, active coping strategies and female sex were associated with less favorable sedation outcomes. Dental practitioners should focus on pre-sedation preparation and parental support, particularly teaching acceptance-based coping strategies, to improve sedation outcomes and overall dental care for children.

## Data Availability

The raw data supporting the conclusions of this article will be made available by the authors, without undue reservation.
